# Pubertal timing and self-harm: a prospective cohort analysis of males and females

**DOI:** 10.1017/S2045796020000839

**Published:** 2020-10-06

**Authors:** Elystan Roberts, Carol Joinson, David Gunnell, Abigail Fraser, Becky Mars

**Affiliations:** 1 National Institute for Health Research Bristol Biomedical Research Centre, University Hospitals Bristol NHS Foundation Trust and University of Bristol, Bristol, UK; 2 Department of Population Health Sciences, University of Bristol, Bristol, UK; 3 Medical Research Council Integrated Epidemiology Unit at the University of Bristol, Bristol, UK

**Keywords:** ALSPAC, self-harm, pubertal timing, puberty

## Abstract

**Aims:**

Early puberty is associated with an increased risk of self-harm in adolescent females but results for males are inconsistent. This may be due to the use of subjective measures of pubertal timing, which may be biased. There is also limited evidence for the persistence of pubertal timing effects beyond adolescence, particularly in males. The primary aim of the current study was therefore to examine the association between pubertal timing and self-harm in both sexes during adolescence and young adulthood, using an objective measure of pubertal timing (age at peak height velocity; aPHV). A secondary aim was to examine whether this association differs for self-harm with *v*. without suicidal intent.

**Methods:**

The sample (*n* = 5369, 47% male) was drawn from the Avon Longitudinal Study of Parents and Children (ALSPAC), a prospective birth cohort study. Mixed-effects growth curve models were used to calculate aPHV. Lifetime history of self-harm was self-reported at age 16 and 21 years, and associated suicidal intent was examined at age 16 years. Associations were estimated using multivariable logistic regression adjusted for a range of confounders. Missing data were imputed using Multiple Imputation by Chained Equations.

**Results:**

Later aPHV was associated with a reduced risk of self-harm at 16 years in both sexes (females: adjusted per-year increase in aPHV OR 0.85; 95% CI 0.75–0.96; males: OR 0.72; 95% CI 0.59–0.88). Associations were similar for self-harm with and without suicidal intent. There was some evidence of an association by age 21 years in females (adjusted per-year increase in aPHV OR 0.91; 95% CI 0.80–1.04), although the findings did not reach conventional levels of significance. There was no evidence of an association by age 21 years in males (adjusted per-year increase in aPHV OR 0.99; 95% CI 0.74–1.31).

**Conclusions:**

Earlier developing adolescents represent a group at increased risk of self-harm. This increased risk attenuates as adolescents transition into adulthood, particularly in males. Future research is needed to identify the modifiable mechanisms underlying the association between pubertal timing and self-harm risk in order to develop interventions to reduce self-harm in adolescence.

## Introduction

Self-harm is one of the strongest predictors of suicide (Hawton *et al*., 2003), which is the second-leading cause of death globally among 15–29 year olds (World Health Organization, 2014). Men are at particularly high risk, being three times more likely than women to die by suicide in the UK (Office for National Statistics, 2018). Identifying the factors associated with self-harm, particularly the unique risk factors experienced by males and females, is therefore important for developing effective interventions.

Adolescence encompasses the transition through puberty and is a period of substantial physical, social and cognitive change. The process of pubertal development is multifaceted, and individuals differ in their timing of pubertal onset, their pubertal tempo (the speed of development) and the synchrony of the development of different pubertal milestones (Berenbaum *et al*., 2015). The majority of existing research has focused on pubertal timing and has shown an association between earlier timing of puberty relative to one's peers and a range of adverse mental health outcomes (e.g. Kaltiala-Heino *et al*., 2003), including self-harm (Patton *et al*., 2007). Few studies, however, have examined potential sex differences in the association between pubertal timing and self-harm. Previous research has reported a consistent association between early pubertal timing and an increased risk of self-harm in females (Roberts *et al*., 2019), but few studies include males or stratify by sex. Those that do find varying results in males, with an increased risk of self-harm having been reported in individuals experiencing early puberty (Michaud *et al*., 2006) and late puberty (Wichstrøm, 2000), or being unrelated to pubertal timing (Graber *et al*., 1997). Drawing conclusions from previous research is further complicated by inadequate adjustment for potential confounding.

The inconsistent findings in males could be due to difficulties in measuring pubertal timing, because none of the previous studies of pubertal timing and self-harm in males have used objective assessments of pubertal timing. In females, age at onset of menarche is an objective, salient indicator of pubertal timing (Dorn and Biro, 2011), but no direct equivalent exists in males: genital development as well as the development of secondary sexual characteristics (pubic and axillary hair growth, voice change) are gradual changes rather than acute events (Marshall and Tanner, 1970). Age at spermarche (the beginning of spermatozoa development in the testicles) is difficult to measure; it requires either urine samples (Kulin *et al*., 1989) or self-report of age at first ejaculation, the validity of which is untested (Dorn *et al*., 2006). Previous research on males' pubertal timing has relied on perceived pubertal timing relative to one's peers (e.g. Wichstrøm, 2000), or inferred pubertal timing from self-reported data on pubertal stage and age (e.g. Patton *et al*., 2007). However, self-reported pubertal stage is subjective and agreement with physical examination tends to be low (Dorn and Biro, 2011). Furthermore, perceived pubertal development compared to peers tends to be biased towards the average as self-perceptions are influenced by social comparison (Alsaker, 1992).

The current study is the first to use an objective measure of pubertal timing in males – age at peak height velocity (aPHV) – to examine its relationship with the risk of self-harm. As individuals progress through adolescence, their height undergoes a sharp rise over a relatively short period of time, known as the adolescent ‘growth spurt’ or peak height velocity. The distribution of aPHV is normal in the general population and correlates well with other measures of pubertal timing (correlation with age at menarche in ALSPAC *r* = 0.79; in previous research *r* = 0.81; Demirjian *et al*., 1985) (Marshall and Tanner, 1969, 1970). aPHV therefore represents a useful proxy for measuring pubertal timing, and using the same exposure variable in males and females allows some comparison of the relative effect of pubertal timing in both sexes. We examine the association between aPHV and lifetime history of self-harm in males and females at ages 16 and 21 years. We also investigate whether findings differ for self-harm with and without suicidal intent, as prior research has indicated there may be differences in the risk factors for these behaviours (Mars *et al*., 2014).

## Methods

### Participants

**Fig. 1.**
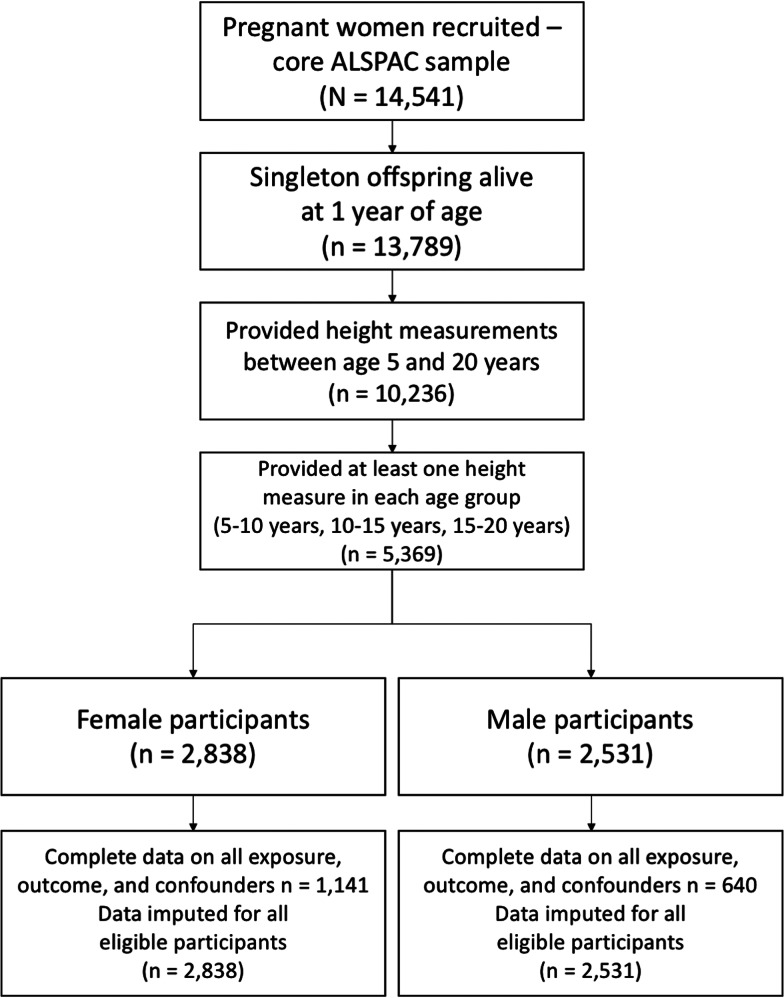
CONSORT flow diagram showing sample derivation for the main analysis sample in ALSPAC.

The study is based on the Avon Longitudinal Study of Parents and Children (ALSPAC), a prospective cohort study conducted in the UK. Pregnant women (*n* = 14 541) with an estimated date of delivery between 1 April 1991 and 31 December 1992 were recruited from the former county of Avon and both the mothers and children have been followed up with regular questionnaires and research clinics (Boyd *et al*., 2013; Fraser *et al*., 2013; Northstone *et al*., 2019). Ethical approval for the study was obtained from the ALSPAC Law and Ethics committee and local research ethics committees, and informed consent for the use of data collected via questionnaires and clinics was obtained from participants following the recommendations of the ALSPAC Ethics and Law Committee at the time. From the initial sample of singletons or first-born twins who were alive at 1 year of age (*n* = 13 789), those with valid aPHV data were eligible for inclusion in the study (*n* = 5369, 47% male; see Fig. 1). Compared to those for whom aPHV data were missing (*n* = 8420), participants with aPHV data (*n* = 5369) were more likely to be white, to be in a higher social class, to have a better educated mother and to have parents who were still a couple by the child's fifth birthday (online Supplementary Tables S1–2).

### Measures

#### Age at peak height velocity

aPHV refers to the adolescent ‘growth spurt’ – the point in time when an individual's height is increasing at the fastest rate. Before the age of 7 years, a random 10% sample of the total cohort were invited to annual or biennial research clinics, but from age 7 years onward (between the ages of 7 and 20 years), all enrolled participants were invited to nine research clinics in which height was measured by research staff using a standard protocol. To calculate growth trajectories over adolescence, the data were restricted to participants who had at least one height measurement recorded during three time periods: age 5 to 10, 10 to <15 and 15 to 20 years. Each participant for whom aPHV was calculated had on average eight height measurements over adolescence (from a maximum of ten) between the ages of 5 and 20 years (Frysz *et al*., 2018). aPHV was derived from these height measurements using Superimposition by Translation and Rotation (SITAR), a mixed-effects growth curve analysis described in detail elsewhere (Frysz *et al*., 2018). Here we also categorised the continuous aPHV variable into normative (the mean aPHV ± 1 standard deviation: 12.7–14.4 years for males; 11.0–12.6 years for females), early (<12.7 years for males; <11.0 years for females) and late (>14.4 years for males; >12.6 years for females) aPHV.

#### Lifetime self-harm

Participants completed self-report questionnaires at ages 16 and 21 years which were based on the Child and Adolescent Self-harm in Europe (CASE) study (Madge *et al*., 2008). They included the question ‘Have you ever hurt yourself on purpose in any way (e.g. by taking an overdose of pills or by cutting yourself)?’. Self-harm was further categorised into suicidal and non-suicidal self-harm (NSSH) based on responses to two follow-up questions. Participants were categorised as having self-harmed with suicidal intent if (i) they responded positively to the question ‘On any of the occasions when you have hurt yourself on purpose, have you ever seriously wanted to kill yourself?’, or (ii) selected ‘I wanted to die’ as a response to the question ‘Do any of the following reasons help to explain why you hurt yourself on that (the most recent) occasion?’.

#### Confounders

Potential confounding variables included in analyses were decided *a priori* based on existing literature. They were: socioeconomic position, measured by material hardship (the difficulty in affording everyday items; Joinson *et al*., 2017) and maternal education level indexed by completed British school examinations and defined as: lower than O-levels (school examinations taken around age 16 in England), O-levels, A-levels (examinations taken around age 18 years) and university degree (Jargowsky and Bane, 1990). Models were also adjusted for parental separation before the child's fifth birthday (reported by mothers; Culpin *et al*., 2014); childhood sexual abuse (retrospectively self-reported at age 22 years; Noll *et al*., 2017); maternal depression during pregnancy (dichotomised at a cut-off score of 12 on the Edinburgh Postnatal Depression Scale (EPDS); Cox *et al*., 1987; Culpin *et al*., 2014); and body mass index (BMI) at age 9 (calculated based on measurements taken at research clinics or self-reported where clinic data were unavailable; Deardorff *et al*., 2011).

#### Statistical analysis

We assessed associations between aPHV and self-harm at ages 16 and 21 years in males and females separately using multivariable logistic regression analyses adjusting for the confounders described above. Secondary analysis examined whether associations differed for self-harm with and without suicidal intent using multinomial logistic regression analyses. All analyses were carried out using Stata version 15.

#### Missing data

There were 5369 participants with aPHV data eligible for inclusion in the study. Complete data on all exposures, outcomes and confounders were available for 1781 participants (36% male; online Supplementary Tables S6–7). Missing outcome and confounder data were imputed using Multiple Imputation by Chained Equations (MICE; Royston and White, 2011); 50 datasets were imputed, and analyses were conducted on these data. Monte Carlo errors are available on request. The use of MICE is based on the Missing at Random (MAR) assumption that conditional on the variables included in the imputation model, there are no systematic differences between observed and missing values for a given variable (Sterne *et al*., 2009). The imputation model incorporated all variables used in the analyses as well as relevant auxiliary variables such as additional measures of socioeconomic status, mental health outcomes and substance use, and earlier or later recordings of variables of interest (online Supplementary Table S3). Missing data were imputed separately by sex to allow for interactions between aPHV and sex in their association with self-harm.

## Results

One in ten males and a quarter of females reported having self-harmed at age 16 years (males 10.8%; females 25.9%). By age 21 years, the proportion of males reporting having self-harmed was 27.8%, and the proportion of females was 35.2%. The final distributions of data in the imputed datasets were similar to the observed data, although there is some evidence of a higher prevalence of self-harm and sexual abuse in the imputed data (online Supplementary Tables S6–7).

Mean aPHV was 13.5 years in males (s.d. = 0.86, range 10.8–16.6 years) and 11.8 years in females (s.d. = 0.82, range 9.1–14.5 years). Table 1 presents the distributions of outcome and confounder variables by aPHV for males and females. In both sexes, the proportion of participants reporting self-harm was highest among those with early aPHV and lowest among those with late aPHV. Those with early aPHV in both sexes had higher BMI at age 9 years, and lower socioeconomic status, than those with normative and late aPHV.

**Table 1.** Distribution of outcome and confounder variables in each category of aPHV timing for both males and females in imputed data

Variable		Distribution					
		Proportion (s.e.) for categorical variables					
		Mean (s.e.) for continuous variables					
		Males (*n* = 2531)			Females (*n* = 2837)		
		Early	Normative	Late	Early	Normative	Late
Self-harm (age 16 years)		15.93 (2.33)	10.38 (1.06)	5.21 (1.62)	28.99 (2.37)	26.26 (1.18)	19.66 (2.05)
Non-suicidal self-harm (age 16 years)		11.13 (1.85)	7.35 (0.87)	3.05 (1.31)	20.63 (2.08)	19.00 (1.02)	12.89 (1.77)
Self-harm with suicidal intent (age 16 years)		4.78 (1.43)	3.03 (0.56)	2.15 (0.94)	8.36 (1.46)	7.26 (0.65)	6.78 (1.32)
Self-harm (age 21 years)		29.51 (4.92)	27.39 (2.57)	28.12 (6.19)	40.15 (2.90)	35.24 (1.54)	29.79 (2.73)
Maternal education	<O-level	18.25 (1.91)	18.72 (0.95)	17.87 (2.06)	22.25 (1.98)	19.35 (0.92)	17.76 (1.84)
	O-level	36.38 (2.40)	33.75 (1.15)	34.45 (2.53)	34.72 (2.27)	35.79 (1.10)	32.34 (2.25)
	A-level	29.11 (2.25)	28.95 (1.09)	29.77 (2.45)	26.28 (2.08)	27.24 (1.02)	30.21 (2.20)
	Degree	16.26 (1.83)	18.59 (0.94)	17.91 (2.04)	16.76 (1.76)	17.62 (0.88)	19.69 (1.90)
Maternal depression		8.35 (1.41)	10.96 (0.80)	10.35 (1.76)	10.55 (1.51)	10.78 (0.76)	8.21 (1.36)
Sexual abuse		4.74 (1.63)	5.46 (1.12)	4.28 (1.81)	7.48 (1.72)	5.67 (0.73)	4.73 (1.41)
Parental separation		13.33 (1.66)	13.15 (0.81)	12.15 (1.72)	15.18 (1.67)	13.46 (7.77)	13.48 (16.21)
Material hardship		2.20 (1.63)	2.01 (0.72)	1.87 (1.52)	2.02 (0.14)	2.01 (0.07)	1.81 (0.14)
Body mass index (BMI)		18.68 (0.16)	17.21 (0.06	16.68 (0.13)	19.67 (0.16)	17.73 (0.06)	16.41 (0.11)

There was weak evidence for a sex interaction in the association between aPHV and self-harm risk at age 16 years (*F* = 3.12, *p* = 0.08). All analyses were stratified by sex, and the results of our main analyses suggested a slightly stronger association in males than in females. We did not find evidence for an interaction with sex at age 21 years (*F* = 4.22, *p* = 0.530).

### Association between pubertal timing and risk of self-harm in males

Table 2 shows the unadjusted and adjusted odds ratios (ORs) and 95% confidence intervals (CIs) for the association between aPHV and self-harm at age 16 and 21 years in males. Later aPHV was associated with reduced risk of self-harm at age 16 years, and findings were consistent following adjustment for confounders (per-year increase in aPHV OR 0.72; 95% CI 0.59–0.88). Compared with the normative timing group, those experiencing early aPHV were at increased risk of self-harm (adjusted OR 1.46; 95% CI 0.98–2.18), whereas those experiencing late aPHV were at reduced risk (adjusted OR 0.49; 95% CI 0.24–0.99). There was no strong evidence of an association with self-harm reported by age 21 years (adjusted per-year increase in aPHV OR 0.99; 95% CI 0.74–1.31), with the results of the categorical analysis also showing no evidence of an association between early or late aPHV (compared to normative) and self-harm risk by age 21 years (Table 2).

**Table 2.** Associations between age at peak height velocity (PHV) and self-harm at age 16 and age 21 years in males

	Age 16				Age 21			
	Unadjusted		Adjusted		Unadjusted		Adjusted	
	OR (95% CI)	*p*	OR (95% CI)	*p*	OR (95% CI)	*p*	OR (95% CI)	*p*
Per one-year increase in aPHV	0.67 (0.56–0.82)	<0.001	0.72 (0.59–0.88)	0.002	0.96 (0.74–1.26)	0.789	0.99 (0.74–1.31)	0.923
Timing of aPHV								
Early (<12.7 years)	1.62 (1.11–2.38)	0.013	1.46 (0.98–2.18)	0.061	1.13 (0.76–1.68)	0.536	1.10 (0.74–1.66)	0.629
Normative (12.7–14.4 years)	1.00	-	1.00	-	1.00	-	1.00	-
Late (>14.4 years)	0.47 (0.23–0.93)	0.032	0.49 (0.24–0.99)	0.045	1.03 (0.65–1.64)	0.899	1.06 (0.66–1.71)	0.813

Model 1 is unadjusted; Model 2 adjusted for maternal education, material hardship, maternal depression, childhood sexual abuse and body mass index (BMI). *N* = 2531.

### Association between pubertal timing and risk of self-harm in females

Unadjusted and adjusted ORs and 95% CIs for the association between aPHV and self-harm reported at age 16 and by age 21 years in females are shown in Table 3. These results are broadly consistent to those found in males, with later aPHV being associated with a lower risk of self-harm at age 16 years in fully adjusted models (adjusted per-year increase in aPHV OR 0.85; 95% CI 0.75–0.96). In the categorical analyses, compared to females with normative aPHV those with late aPHV experienced a reduced risk of self-harm (adjusted OR 0.73; 95% CI 0.54–0.97), however there was little evidence for an increased risk in those who experienced early aPHV (adjusted OR 1.07; 95% CI 0.83–1.38). The results by age 21 years were consistent with the age 16 years results (unadjusted per-year increase in aPHV OR 0.86; 95% CI 0.76–0.96), but attenuated after adjustment for confounders (adjusted per-year increase in aPHV OR 0.91; 95% CI 0.80–1.04). In unadjusted categorical analyses, females with early compared with normative aPHV had an increased risk of self-harm (unadjusted OR 1.23, 95% CI 0.97–1.56), and females with late compared with normative aPHV had a reduced risk (unadjusted OR 0.79, 95% CI 0.61–1.02) by age 21 years. However, these associations also attenuated after adjustment for confounders (Table 3).

**Table 3.** Associations between age at peak height velocity (PHV) and self-harm at age 16 and 21 years in females

	Age 16				Age 21			
	Unadjusted		Adjusted		Unadjusted		Adjusted	
	OR (95% CI)	*p*	OR (95% CI)	*p*	OR (95% CI)	*p*	OR (95% CI)	*p*
Per one-year increase in aPHV	0.81 (0.72–0.91)	<0.001	0.85 (0.75–0.96)	0.008	0.86 (0.76–0.96)	0.010	0.91 (0.80–1.04)	0.160
Timing of aPHV								
Early (<11.0 years)	1.16 (0.91–1.48)	0.242	1.07 (0.83–1.38)	0.625	1.23 (0.97–1.56)	0.082	1.12 (0.88–1.44)	0.361
Normative (11.0–12.6 years)	1.00	–	1.00	–	1.00	–	1.00	–
Late (>12.6 years)	0.69 (0.51–0.92)	0.010	0.73 (0.54–0.97)	0.032	0.79 (0.61–1.02)	0.070	0.85 (0.65–1.11)	0.222

Model 1 is unadjusted; Model 2 adjusted for maternal education, material hardship, maternal depression, childhood sexual abuse and body mass index (BMI). *N* = 2838.

### Secondary analysis

We examined associations between aPHV and self-harm with *v*. without suicidal intent at age 16 years. There was no strong evidence in either sex to suggest that associations between aPHV and self-harm differ according to suicidal intent (males: adjusted OR 1.05, 95% CI 0.72–1.54; females: adjusted OR 1.11, 95% CI 0.89–1.40; online Supplementary Tables S4–5). The overall pattern of results was consistent across analyses in the complete case and imputed datasets (online Supplementary Tables S8–9).

## Discussion

We found evidence for an inverse association between aPHV and lifetime risk of self-harm in both males and females at age 16 years. There was no strong evidence of a difference in the associations between aPHV and NSSH *v*. self-harm with suicidal intent. There was weak evidence for an association between aPHV and self-harm by 21 years in females but not males; however, there was no formal statistical evidence of a sex difference in the association.

### Strengths and limitations

This study has a number of strengths, including the large, prospective, community cohort sample. A community sample is important as most episodes of self-harm do not present to medical services (Kidger *et al*., 2012). We also adjusted for a number of potential confounding factors, although some residual confounding may remain. Another major strength and novel feature of our study is the use of aPHV as an objective measure of pubertal timing. aPHV correlates well with other measures of pubertal timing and measuring it in both sexes allowed us to examine sex differences in the relationship between pubertal timing and risk of self-harm. However, peak height velocity occurs earlier in puberty in females (around Tanner pubic hair stage three) than in males (around Tanner pubic hair stage four; Marshall and Tanner, 1969, 1970); males may have therefore experienced more pubertal milestones before peak height velocity than females, and this difference in pubertal stage could influence its association with self-harm. Our aPHV variable was based on direct measures of the participants' height, recorded by trained professionals across childhood and adolescence, reducing the possibility of measurement error in the exposure variable. However, there were differences between those with and without aPHV data (online Supplementary Tables S1–2), and it is possible that restricting the sample to individuals who attended multiple research clinics may limit the generalisability of the results. We also examined self-harm data at two time points, allowing us to investigate whether the association between pubertal timing and self-harm persists from adolescence into early adulthood.

This study must also be interpreted in the context of its limitations. It is widely accepted that the gold standard measurement of pubertal development is physician examination using the Tanner Scale (Marshall and Tanner, 1969, 1970). However, this method was not feasible for a large-scale prospective study. Although self-reported Tanner stages could have been examined, they are often unreliable: in ALSPAC over a quarter (27%) of males reported regression in genital Tanner stage from one time point to the next (Monteilh *et al*., 2011). Further, the height measurements which were used to calculate aPHV would ideally have been taken more frequently (e.g. every 6 months) to capture growth trajectories with maximum accuracy; unfortunately, this was not possible within the funding constraints of a large cohort study.

Loss to follow-up affects most cohort studies and can introduce bias. To address this issue, we imputed missing data up to the number of individuals with valid aPHV data (*n* = 5369). The wide range of data available in ALSPAC permitted the inclusion of many relevant auxiliary variables in the imputation model, which maximises confidence in the MAR assumption. A further potential limitation is unreliability in the self-harm variable, which was assessed via self-report. For the derivation of the lifetime self-harm by age 21 years variable, participants were coded as having self-harmed if they had responded positively at either timepoint; however, over a quarter of the participants who reported lifetime self-harm at age 16 years (28.24% males, 28.17% females) reported no lifetime self-harm at age 21 years. It is unclear whether these individuals represent false positives at age 16 years or false negatives at age 21 years. Participants at age 21 years may have forgotten or reappraised earlier self-harm (Plener *et al*., 2015). Nevertheless, sensitivity analyses using only participants who reported self-harm at age 21 years, and only participants who provided consistent reports at age 16 and 21 years, showed similar results (albeit with weaker evidence; online Supplementary Tables S10–11). We also cannot rule out the possibility that for some individuals, lifetime self-harm may have occurred before aPHV. However, pre-pubertal self-harm incidence is low (Whitlock and Selekman, 2014).

### Comparison with existing literature

The current finding of an association between earlier pubertal timing (indicated by earlier aPHV) and an increased risk of self-harm at age 16 years is consistent with earlier research investigating the association between pubertal timing and self-harm in females (Roberts *et al*., 2019), and some studies that have examined pubertal timing and self-harm in males (e.g. Michaud *et al*., 2006). Our results are consistent with the *early timing hypothesis* (Brooks-Gunn and Warren, 1985; Ge and Natsuaki, 2009), which proposes that early developers are at the greatest risk for adverse mental health outcomes during adolescence. This could be because they are more likely to associate with older peers, and as a result are exposed at a younger age to risky behaviours (Stattin and Magnusson, 1990) which may increase self-harm risk (Patton *et al*., 2007). Early developers may also be less prepared for physical maturity and its associated social implications than their normative- and late-maturing peers. This may be due to experiencing a shorter period before the onset of puberty to acquire necessary skills to manage the challenges of the pubertal transition (Ge *et al*., 2003). An associated contributing mechanism may be an increased feeling of isolation: early developers begin a new pubertal phase of life, with its accompanying stress and uncertainty, without the support of same-age peers going through similar experiences (Ge and Natsuaki, 2009).

Biological and neurocognitive mechanisms have also been proposed to explain the relationship between early pubertal timing and adverse psychological outcomes. For example, dopaminergic systems (associated with sensation-seeking) mature with advancing pubertal development, while executive function systems in the prefrontal cortex (associated with planning and inhibition) develop with advancing chronological age. This mismatch in development of sensation-seeking and inhibitory function might lead to more risky behaviours including self-harm (Steinberg, 2008).

There may be sex-specific mechanisms underlying the association between earlier puberty and self-harm. Some effects may be unique or stronger for females; for example, the increased risk of self-harm in earlier developers may be a result of early experiences of sexualisation and harassment (Skoog *et al*., 2016), or body dissatisfaction arising from the development of body shape that deviates from a perceived thin ideal (Berger *et al*., 2009). Conversely, in males, transitioning into puberty is associated with endorsing typically ‘masculine’ traits such as independence and self-reliance (Galambos *et al*., 1990). Males who experience early aPHV may feel pressured to become more independent before they develop the cognitive and emotional capacity to do so.

However, our findings differ to those of other studies, particularly in males, that have reported an association between late pubertal timing and an increased risk of self-harm (e.g. Wichstrøm, 2000). An adverse effect of late puberty in males may be related to bullying, since victims of bullying tend to be small and weak for their age (Olweus, 1991), perhaps due to later pubertal development. These findings broadly support the *maturational deviance hypothesis*, which postulates that adolescents who mature at different ages to the population norm, whether early or late, are at increased risk of adverse psychological effects (Alsaker, 1992).

We did not find any evidence that the association between aPHV and self-harm differs according to whether or not self-harm is accompanied by suicidal intent. This result is consistent with previous research (Larsson and Sund, 2008; Roberts *et al*., 2019) and implies that although some risk factors differ for suicidal and NSSH (Mars *et al*., 2014), pubertal timing is one of the many risk factors shared by both. Our results also provide no evidence that adverse effects of early pubertal timing persist into early adulthood in males, but some weak evidence that associations persist in females. However, there was no statistical evidence of a sex interaction so sex differences must be interpreted with caution. Transient pubertal timing effects are consistent with previous literature (Senia *et al*., 2018). However, in an existing study analysing females in the same cohort (Roberts *et al*., 2019) and using age at menarche as an indicator of pubertal timing, there was evidence for a persistent effect of early menarche on the risk of self-harm by age 21 years (adjusted OR per-year increase in age at menarche 0.92; 95% CI 0.85–1.00). The difference in the strength of the association may be due to the different samples included in each study: nearly twice as many females in ALSPAC (*n* = 4049) provided data on age at menarche than provided aPHV data, so the current study has lower female-specific statistical power. The different measures of pubertal timing may also have affected the results, for example, by reflecting different hormonal mechanisms that may have different effects on psychological outcomes (Dorn and Biro, 2011). Nevertheless, the effect estimates in both studies are consistent, differing only in their level of confidence. It has been hypothesised that early pubertal timing increases adolescents' exposure to ‘snares’: experiences which occur during adolescence but have long-term adverse effects (Moffitt *et al*., 2002). Female adolescents may be particularly adversely affected by some of these snares, for example, teenage pregnancy. However, we did not find statistical evidence for a sex difference in the association by age 21 years; more research is needed to investigate whether early pubertal timing effects on risk of self-harm are more persistent in females than in males.

## Conclusions

The current study provides evidence of an association between earlier pubertal timing and an increased risk of self-harm in both males and females in adolescence. The association attenuated by age 21 years. Future research is needed to determine whether this association is causal; to identify underlying mechanisms, and to examine whether these mechanisms differ by sex. Identification of translational targets for intervention may help to inform the development of interventions to reduce the risk of self-harm in adolescence.

## Data Availability

Individual-level ALSPAC data are available following an application. This process of managed access is detailed at www.bristol.ac.uk/alspac/researchers/access. Cohort details and data descriptions for ALSPAC are publicly available at the same web address.

## Supplementary material

For supplementary material accompanying this paper visit https://doi.org/10.1017/S2045796020000839. click here to view supplementary material
