# Supplementary material for: Pubertal timing and self-harm: a prospective cohort analysis of males and females
Source: Epidemiol Psychiatr Sci. 2020 Oct 6;29:e170. doi: 10.1017/S2045796020000839 (PMC7576520; doi:10.1017/S2045796020000839)
Supplement: Supplementary file 1 [file S2045796020000839sup001.docx]

**Supplementary Table 1** Observed values of descriptive data in males who did and did not provide data on age at peak height velocity in the core ALSPAC sample.

| Variable | Description | N | Data on age at PHV  (n = 2,531) | No data on age at PHV  (n = 4,584) | X^2^ | P |
| --- | --- | --- | --- | --- | --- | --- |
| Self-harm at age 16 | Yes | 177 | 140  (9.27%) | 37  (8.10%) | 0.59 | .442 |
|  | No | 1,790 | 1,370  (90.73%) | 420  (91.90% |  |  |
| Maternal education level | < O level | 1,921 | 450  (18.26%) | 1,471  (38.19%) | 386.95 | <.001 |
|  | O level | 2,188 | 843  (34.21%) | 1,345  (34.92%) |  |  |
|  | A level | 1,414 | 720  (29.22%) | 694  (18.02%) |  |  |
|  | Degree or higher | 793 | 451  (18.30%) | 342  (8.88%) |  |  |
| Material hardship category | <5 | 3,708 | 1,916  (90.68%) | 1,792  (88.32%) | 8.77 | .012 |
|  | 6-10 | 344 | 163  (7.71%) | 181  (8.92%) |  |  |
|  | 11-15 | 90 | 34  (1.61%) | 56  (2.76%) |  |  |
| Highest parental social class | Professional/managerial | 3,210 | 1,533  (65.18%) | 1,677  (48.34%) | 160.62 | <.001 |
|  | Other | 2,611 | 819  (34.82%) | 1,792  (51.66%) |  |  |
| Parental separation before child’s 5^th^ birthday | Yes | 1,210 | 330  (13.04%) | 880  (19.20%) | 43.82 | <.001 |
|  | No | 5,905 | 2,201  (86.96%) | 3,704  (80.80%) |  |  |
| Child’s ethnicity | White | 6,101 | 2,414  (98.33) | 3,687  (96.67%) | 15.78 | <.001 |
|  | Other | 168 | 41  (1.67%) | 127  (3.33%) |  |  |

**Supplementary Table 2** Observed values of descriptive data in females who did and did not provide data on age at peak height velocity in the core ALSPAC sample.

| Variable | Description | N | Data on age at PHV  (n = 2,838) | No data on age at PHV  (n = 3,836) | X^2^ | P |
| --- | --- | --- | --- | --- | --- | --- |
| Self-harm at age 16 | Yes | 718 | 537  (25.69%) | 181  (24.49%) | 0.42 | .519 |
|  | No | 2,111 | 1,553  (74.31%) | 558  (75.51%) |  |  |
| Maternal education level | < O level | 1,760 | 526  (19.11%) | 1,234  (38.77%) | 344.81 | <.001 |
|  | O level | 2,051 | 966  (35.09%) | 1,085  (34.09%) |  |  |
|  | A level | 1,343 | 765  (27.79%) | 578  (18.16%) |  |  |
|  | Degree or higher | 782 | 496  (18.02%) | 286  (8.99%) |  |  |
| Material hardship category | <5 | 3,433 | 2,069  (90.55%) | 1,364  (87.38%) | 9.94 | .007 |
|  | 6-10 | 329 | 174  (7.61%) | 155  (9.93%) |  |  |
|  | 11-15 | 84 | 42  (1.84%) | 42  (2.69%) |  |  |
| Highest parental social class | Professional/managerial | 3,039 | 1,648  (62.09%) | 1,391  (48.45%) | 103.75 | <.001 |
|  | Other | 2,486 | 1,006  (37.91%) | 1,480  (51.55%) |  |  |
| Parental separation before child’s 5^th^ birthday | Yes | 1,092 | 390  (13.74%) | 702  (18.30%) | 24.77 | <.001 |
|  | No | 5,582 | 2,448  (86.26%) | 3,134  (81.70%) |  |  |
| Child’s ethnicity | White | 5,746 | 2,693  (98.00%) | 3,053  (97.01%) | 5.78 | .016 |
|  | Other | 149 | 55  (2.00%) | 94  (2.99%) |  |  |

**Supplementary Table 3** – Multiple Imputation Model

All participants with data on age at peak height velocity were included in analyses. Outcome and covariate data were imputed. The imputation model included all variables used in the analysis (exposures, outcomes, and confounders), in addition to auxiliary variables listed below. Bespoke combinations of auxiliary data, specific to each imputation model, were utilised.

| Variable name | Details | Type of variable |
| --- | --- | --- |
| **Demographic variables** |  |  |
| Crowding | Number of individuals in the household >4; collected at 8 weeks’ gestation | Categorical |
| Parity | Number of siblings of study child; collected at 18 weeks’ gestation | Continuous |
| Social class | Categorised based on parents’ combined level of social class on the Registrar General’s scale; collected at 32 weeks’ gestation | Ordered categorical (4 categories) |
| Child IQ | Based on the Wechsler Intelligence Scale for Children (WISC); collected at 8 years at a research clinic | Continuous |
| Family adversity | Including measures of early parenthood, housing adequacy, and partner cruelty; collected up to child age 4 years at research clinics and using questionnaires | Categorical |
| **Family mental health** |  |  |
| Maternal suicide attempt | Collected up until age 9 years using questionnaires | Categorical |
| Maternal depression | Collected at child age 8 weeks and 21 years old using the Edinburgh Postnatal Depression Scale | Continuous |
| Exposure to family self-harm | Child-reported, collected at age 16 using a questionnaire | Categorical |
| Exposure to maternal self-harm | Child-reported, collected at age 16 using a questionnaire | Categorical |
| **Parent substance use** |  |  |
| Maternal cannabis use | Mother-reported, collected at child age 7 and 9 years | Categorical |
| **Child mental health** |  |  |
| Child depressive disorder | Collected at age 15 years using the Development and Wellbeing Assessment (DAWBA) scale and at age 18 years using the Clinical Interview Schedule – Revised (CISR) | Categorical |
| Child anxiety disorder | Collected at age 15 years using the Development and Wellbeing Assessment (DAWBA) scale and at age 18 years using the Clinical Interview Schedule – Revised (CISR) | Categorical |
| Child depressive symptoms | Collected at age 10, 12, 13, 16, 17, and 18 years using the Mood and Feelings Questionnaire (MFQ) | Categorical |
| **Child substance use** |  |  |
| Child smoking | Collected at age 13, 16, and 17 years at research clinics and at age 14 years using a questionnaire | Categorical |
| Child heavy alcohol use | Collected at age 13 and 16 years at research clinics | Categorical |
| Child cannabis use | Collected at age 13, 16, and 17 years at research clinics | Categorical |
| Child illicit drug use | Collected at age 16 years at a research clinic and using a questionnaire | Categorical |
| **Previous self-harm** |  |  |
| Lifetime self-harm | Collected at age 11, 15, and 18 years at research clinics | Categorical |

**Supplementary Table 4** Associations between age at peak height velocity (aPHV) and suicidal and non-suicidal self-harm, versus no self-harm, as well as suicidal versus non-suicidal self-harm, at age 16 in males. Adjusted results are adjusted for maternal education, material hardship, maternal depression, childhood sexual abuse, and body mass index (BMI). N = 2,531

|  | **Non-suicidal self-harm v no self-harm** | | | | **Suicidal self-harm v no self-harm** | | | | **Suicidal v non-suicidal self-harm** | | | |
| --- | --- | --- | --- | --- | --- | --- | --- | --- | --- | --- | --- | --- |
|  | Unadjusted | | Adjusted | | Unadjusted | | Adjusted | | Unadjusted | | Adjusted | |
|  | RRR (95% CI) | p | RRR (95% CI) | p | RRR (95% CI) | p | RRR (95% CI) | p | RRR (95% CI) | p | RRR (95% CI) | p |
| Per one-year increase in aPHV | 0.65  (0.52 – 0.82) | <.001 | 0.71  (0.56 – 0.90) | .005 | 0.73  (0.54 – 0.99) | .040 | 0.74  (0.54 – 1.03) | .072 | 1.11  (0.77 – 1.60) | .557 | 1.05  (0.72 – 1.54) | .786 |
| Timing of aPHV | | | | | | | | | | | | |
| Early  (<12.7 years) | 1.61  (1.03 – 2.52) | .038 | 1.41  (0.88 – 2.25) | .153 | 1.65  (0.86 – 3.14) | .130 | 1.59  (0.81 – 3.10) | .176 | 1.02  (0.48 – 2.18) | .954 | 1.13  (0.51 – 2.47) | .764 |
| Normative  (12.7-14.4 years) | 1.00 | - | 1.00 | - | 1.00 | - | 1.00 | - | 1.00 | - | 1.00 | - |
| Late  (>14.4 years) | 0.38  (0.15 – 0.94) | .036 | 0.40  (0.16 – 1.00) | .049 | 0.66  (0.26 – 1.68) | .379 | 0.67  (0.26 – 1.73) | .411 | 1.73  (0.52 – 5.79) | .375 | 1.68  (0.50 – 5.64) | .402 |

**Supplementary Table 5** Associations between age at peak height velocity (PHV) and suicidal and non-suicidal self-harm, versus no self-harm, as well as suicidal versus non-suicidal self-harm, at age 16 in females. Adjusted results are adjusted for maternal education, material hardship, maternal depression, childhood sexual abuse, and body mass index (BMI). N = 2,838

|  | **Non-suicidal self-harm v no self-harm** | | | | **Suicidal self-harm v no self-harm** | | | | **Suicidal v non-suicidal self-harm** | | | |
| --- | --- | --- | --- | --- | --- | --- | --- | --- | --- | --- | --- | --- |
|  | Unadjusted | | Adjusted | | Unadjusted | | Adjusted | | Unadjusted | | Adjusted | |
|  | RRR (95% CI) | p | RRR (95% CI) | p | RRR (95% CI) | p | RRR (95% CI) | p | RRR (95% CI) | p | RRR (95% CI) | p |
| Per one-year increase in aPHV | 0.78  (0.69 – 0.89) | <.001 | 0.82  (0.71 – 0.94) | .006 | 0.88  (0.72 – 1.06) | .176 | 0.91  (0.74 – 1.12) | .387 | 1.12  (0.90 – 1.38) | .304 | 1.11  (0.89 – 1.40) | .359 |
| Timing of aPHV | | | | | | | | | | | | |
| Early  (<11.0 years) | 1.14  (0.87 – 1.50) | .350 | 1.04  (0.78 – 1.39) | .779 | 1.20  (0.79 – 1.81) | .394 | 1.13  (0.73 – 1.74) | .592 | 1.05  (0.66 – 1.66) | .839 | 1.08  (0.67 – 1.75) | .753 |
| Normative  (11.0-12.6 years) | 1.00 | - | 1.00 | - | 1.00 | - | 1.00 | - | 1.00 | - | 1.00 | - |
| Late  (>12.6 years) | 0.62  (0.44 – 0.87) | .006 | 0.66  (0.47 – 0.93) | .016 | 0.85  (0.54 – 1.35) | .487 | 0.90  (0.56 – 1.44) | .657 | 1.37  (0.80 – 2.33) | .238 | 1.37  (0.80 – 2.34) | .255 |

**Supplementary Table 6** Distributions of values of exposure, outcome, and confounder variables observed in males with complete data for all included variables, and distributions in imputed datasets. Imputed data *n* = 2,531; *n*s in the observed data rows differ according to the amount of missing data.

| Imputed variable | | | n (%) data missing | Distribution  n (%) for categorical variables  Mean (SE) for continuous variables | |
| --- | --- | --- | --- | --- | --- |
|  | | |  | Observed data | Imputed datasets |
| Timing of aPHV | Early | | 0 | 420 / 2,531  (16.59) | 16.59 |
|  | Normative | | 0 | 1,749 / 2,531  (69.10) | 69.10 |
|  | Late | | 0 | 362 / 2,531  (14.30) | 14.30 |
| Self-harm (age 16 years) | | | 1,021  (40.34) | 140 / 1,510  (9.27) | 10.85 |
| Non-suicidal self-harm (age 16 years) | | |  | 98 / 1,510  (6.49) | 7.65 |
| Self-harm with suicidal intent (age 16 years) | | |  | 42 / 1,510  (2.78) | 3.20 |
| Self-harm (age 21 years) | | | 1,353  (53.46) | 150 / 1,178  (12.73) | 15.63 |
| Maternal education | | < O-level | 67  (2.65) | 450 / 2,464  (18.26) | 18.51 |
|  |  | O-level |  | 843 / 2,464  (34.21) | 34.29 |
|  |  | A-level |  | 720 / 2,464  (29.22) | 29.09 |
|  |  | Degree |  | 451 / 2,464  (18.30) | 18.12 |
| Maternal depression | | | 256  (10.11) | 227 / 2,275  (9.98) | 10.50 |
| Sexual abuse | | | 1,546  (61.08) | 14 / 985  (1.42) | 5.20 |
| Parental separation | | | - | 330 / 2,531  (13.04) | 13.04 |
| Material hardship | | | - | 1.87  (2.68) | 2.04  (0.06) |
| Body mass index (BMI) | | | - | 17.38  (2.63) | 17.38  (0.05) |

**Supplementary Table 7** Distributions of values of exposure, outcome, and confounder variables observed in females with complete data for all included variables, and distributions in imputed datasets. Imputed data *n* = 2,838; *n*s in the observed data rows differ according to the amount of missing data.

| Imputed variable | | | n (%) data missing | Distribution  n (%) for categorical variables  Mean (SE) for continuous variables | |
| --- | --- | --- | --- | --- | --- |
|  | | |  | Observed data | Imputed datasets |
| Timing of aPHV | Early | | 0 | 461 / 2,838  (16.24) | 16.24 |
|  | Normative | | 0 | 1,932 / 2,838  (68.08) | 68.08 |
|  | Late | | 0 | 445 / 2,838  (15.68) | 15.68 |
| Self-harm (age 16 years) | | | 748  (26.36) | 537 / 2,090  (25.69) | 25.77 |
| Non-suicidal self-harm (age 16 years) | | |  | 384 / 2,090  (18.37) | 18.41 |
| Self-harm with suicidal intent (age 16 years) | | |  | 153 / 2,090  (7.32) | 7.36 |
| Self-harm (age 21 years) | | | 1,001  (35.27) | 494 / 1,837  (26.89) | 27.60 |
| Maternal education | | < O-level | 85  (3.00) | 526 / 2,753  (19.11) | 19.60 |
|  |  | O-level |  | 966 / 2,753  (35.09) | 35.08 |
|  |  | A-level |  | 765 / 2,753  (27.79) | 27.53 |
|  |  | Degree |  | 496 / 2,753  (18.02) | 17.79 |
| Maternal depression | | | 322  (11.35) | 252 / 2,516  (10.02) | 10.33 |
| Sexual abuse | | | 1,097  (38.65) | 75 / 1,741  (4.31) | 6.03 |
| Parental separation | | | - | 390 / 2,838  (13.74) | 13.74 |
| Material hardship | | | - | 1.80  (2.68) | 1.99  (0.06) |
| Body mass index (BMI) | | | - | 17.83  (2.92) | 17.83  (0.06) |

**Supplementary Table 8** Associations between age at peak height velocity (PHV) and self-harm in complete case data at age 16 and age 21 years in males. All models adjusted for maternal education, material hardship, maternal depression, childhood sexual abuse, and body mass index (BMI). N = 546

Note: analysis for males in the *Late* timing of aPHV category at age 16 was unavailable in the complete case data due to small cell counts.

|  | Age 16 | | | | Age 21 | | | |
| --- | --- | --- | --- | --- | --- | --- | --- | --- |
|  | Unadjusted  OR (95% CI) | p | Adjusted  OR (95% CI) | p | Unadjusted  OR (95% CI) | p | Adjusted  OR (95% CI) | p |
| Per one-year increase in aPHV | 0.62  (0.44 – 0.88) | .008 | 0.67  (0.46 – 0.97) | .033 | 0.79  (0.60 – 1.04) | .094 | 0.84  (0.63 – 1.13) | .244 |
| Timing of aPHV | | | | | | | | |
| *Early*  *(<12.7 years)* | 1.81  (0.99 – 3.31) | .056 | 1.76  (0.93 – 3.33) | .084 | 1.42  (0.83 – 2.43) | .199 | 1.33  (0.76 – 2.33) | .311 |
| *Normative*  *(12.7-14.4 years)* | 1.00 | - | 1.00 | - | 1.00 | - | 1.00 | - |
| *Late*  *(>14.4 years)* | - | - | - | - | 0.57  (0.22 – 1.48) | .245 | 0.61  (0.23 – 1.61) | .315 |

**Supplementary Table 9** Associations between age at peak height velocity (PHV) and self-harm in complete case data at age 16 and age 21 years in females. All models adjusted for maternal education, material hardship, maternal depression, childhood sexual abuse, and body mass index (BMI). N = 1,027

|  | Age 16 | | | | Age 21 | | | |
| --- | --- | --- | --- | --- | --- | --- | --- | --- |
|  | Unadjusted  OR (95% CI) | p | Adjusted  OR (95% CI) | p | Unadjusted  OR (95% CI) | p | Adjusted  OR (95% CI) | p |
| Per one-year increase in aPHV | 0.83  (0.70 – 0.99) | .033 | 0.87  (0.72 – 1.05) | .135 | 0.87  (0.74 – 1.02) | .087 | 0.93  (0.78 – 1.10) | .386 |
| Timing of aPHV | | | | | | | | |
| *Early*  *(<11.0 years)* | 1.25  (0.85 – 1.81) | .258 | 1.13  (0.76 – 1.68) | .551 | 1.38  (0.96 – 1.96) | .079 | 1.24  (0.85 – 1.80) | .257 |
| *Normative*  *(11.0-12.6 years)* | 1.00 | - | 1.00 | - | 1.00 | - | 1.00 | - |
| *Late*  *(>12.6 years)* | 0.79  (0.52 – 1.19) | .257 | 0.84  (0.55 – 1.28) | .427 | 0.90  (0.62 – 1.31) | .592 | 0.98  (0.67 – 1.43) | .909 |

**Supplementary Table 10** Odds ratios showing associations between age at menarche and self-harm reported at age 21 years in males and females. Analysis conducted on the imputed data (n = 5,369).

Note: males aPHV timing: early <12.7 years; normative 12.7-14.4 years; late >14.4 years; females aPHV timing: early <11.0 years; normative 11.0-12.6 years; late >12.6 years.

|  | Males | | | | Females | | | |
| --- | --- | --- | --- | --- | --- | --- | --- | --- |
|  | Complete case OR (95% CI) | p | Imputed datasets OR (95% CI) | p | Complete case OR (95% CI) | p | Imputed datasets OR (95% CI) | p |
| Per one-year increase in aPHV | 1.08  (0.80 – 1.45) | .618 | 1.05  (0.85 – 1.29) | .657 | 0.90  (0.75 – 1.08) | .242 | 0.96  (0.84 – 1.09) | .527 |
| Timing of aPHV | | | | | | | | |
| *Early* | 1.00  (0.54 – 1.86) | .999 | 1.04  (0.70 – 1.53) | .860 | 1.49  (1.02 – 2.18) | .040 | 1.18  (0.91 – 1.53) | .212 |
| *Normative* | 1.00 | - | 1.00 | - | 1.00 | - | 1.00 | - |
| *Late* | 1.23  (0.57 – 2.65) | .601 | 1.13  (0.73 – 1.75) | .595 | 0.97  (0.65 – 1.44) | .876 | 0.93  (0.70 – 1.24) | .615 |

**Supplementary Table 11** Odds ratios showing associations between age at menarche and self-harm reported by age 21 years in males and females who provided consistent reports of either self-harm or no self-harm at age 16 and age 21 years.

Note: males aPHV timing: early <12.7 years; normative 12.7-14.4 years; late >14.4 years; females aPHV timing: early <11.0 years; normative 11.0-12.6 years; late >12.6 years. These analyses were only available on the complete case data (n = 1,573). Analysis for males in the *Late* timing of aPHV category was unavailable due to small cell counts.

|  | Males | | | | Females | | | |
| --- | --- | --- | --- | --- | --- | --- | --- | --- |
|  | Unadjusted  OR (95% CI) | p | Adjusted  OR (95% CI) | p | Unadjusted  OR (95% CI) | p | Adjusted  OR (95% CI) | p |
| Per one-year increase in aPHV | 0.70  (0.46, 1.08) | .105 | 0.78  (0.50, 1.22) | .274 | 0.80  (0.64, 0.97) | .027 | 0.80  (0.64, 1.01) | .064 |
| Timing of aPHV | | | | | | | | |
| *Early* | 1.51  (0.70, 3.26) | .297 | 1.35  (0.60, 3.07) | .471 | 1.54  (0.99, 2.40) | .058 | 1.44  (0.90, 2.29) | .129 |
| *Normative* | 1.00 | - | 1.00 | - | 1.00 | - | 1.00 | - |
| *Late* | - | - | - |  | 0.84  (0.51, 1.38) | .486 | 0.86  (0.51, 1.45) | .574 |
